# Cluster Formation Induced by Local Dielectric Saturation in Restricted Primitive Model Electrolytes

**DOI:** 10.1021/acs.jpclett.4c01829

**Published:** 2024-08-07

**Authors:** David Ribar, Clifford E. Woodward, Sture Nordholm, Jan Forsman

**Affiliations:** † Computational Chemistry, Lund University, P.O. Box 124, S-221 00 Lund, Sweden; ‡ School of Physical, Environmental and Mathematical Sciences, University College, University of New South Wales, ADFA Canberra ACT 2600, Australia; ¶ Department of Chemistry and Molecular Biology, The University of Gothenburg, 412 96 Gothenburg, Sweden

## Abstract

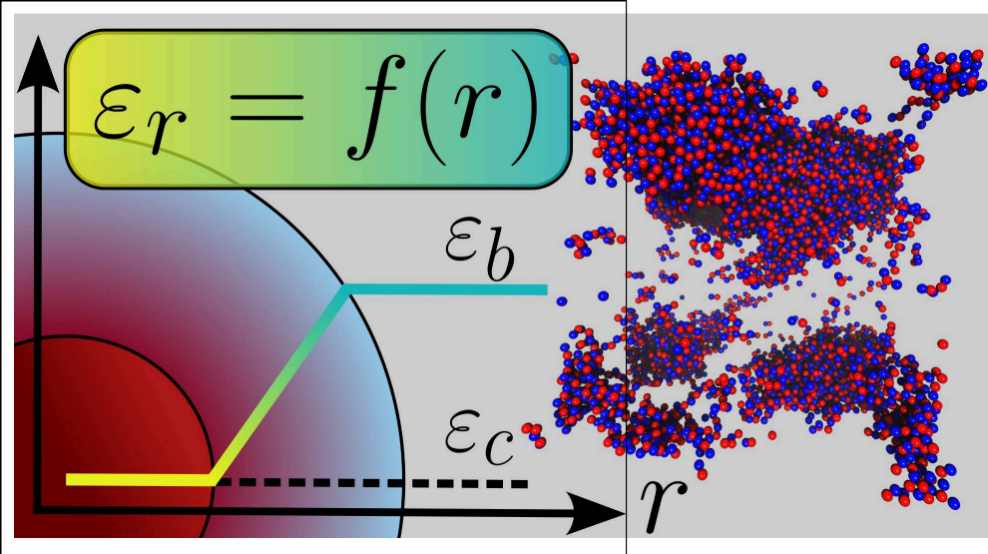

Experiments using the Surface Force Apparatus (SFA) have found anomalously long-ranged charge–charge underscreening in concentrated salt solutions. Meanwhile, theory and simulations have suggested ion clustering to be a possible origin of this behavior. The popular Restricted Primitive Model of electrolyte solutions, in which the solvent is represented by a uniform relative dielectric constant, ε_*r*_, is unable to resolve the anomalous underscreening seen in experiments. In this work, we modify the Restricted Primitive Model to account for local dielectric saturation within the ion hydration shell. The dielectric “constant” in our model locally decreases from the bulk value to a lower saturated value at the ionic surface. The parameters for the model are deduced so that typical salt solubilities are obtained. Our simulations for both bulk and slit geometries show that our model displays strong cluster formation and these give rise to long-ranged density correlations between charged surfaces, at distances similar to what has been observed in SFA measurements. An electrolyte model wherein the dielectric constant remains uniform does not display similar clusters, even with ε_*r*_ equal to the low saturated value at ion contact. Hence, the observed behaviors are not simply due to an enhanced Coulomb interaction. In the latter case, cluster growth is counteracted by long-ranged repulsions between like-charged ions within clusters; this is an effect that is considerably reduced when the dielectric response drop is local. Our results imply that long-ranged interactions in these systems are mainly due to cluster–cluster correlations, rather than large electrostatic screening lengths.

Electrolytes play an important role in a plethora of both scientific and industrial applications.^1−3^ Simple theoretical descriptions at the mean-field level^1,4,5^ have proven to be reasonably accurate for aqueous electrolytes at low coupling strength, e.g., monovalent ions (1:1 salts) in aqueous solvents at low and intermediate concentrations. Perhaps the most fundamental prediction by these theories is *ionic screening*, often described in terms of the so-called Debye screening length, λ_*D*_. The Debye length is predicted to be inversely proportional to the square root of the electrolyte concentration, λ_*D*_ ≈ 1/*c*^1/2^. Salts composed of ions where at least one component is multivalent will generally require a higher level of theory, accounting for ion–ion correlations.^6−9^ This notwithstanding, it was believed that mean-field theories could capture the qualitative behaviors of even concentrated aqueous solutions of 1:1 salts, with some predictable corrections due to correlations. This has been called into question by recent experimental investigations of 1:1 electrolyte solutions, above a threshold concentration of about 1 M. These have revealed a peculiar anomalous underscreening phenomenon^10−15^ whereby the interaction between charged surfaces exhibit an exponential decay with an extraordinarily long-range correlation length, λ, much larger than the Debye length predicted by mean-field theory. Moreover, λ appears to *increase* with the salt concentration, in qualitative disagreement with more-sophisticated theories that attempt to correct for correlations.^16−18^ It should be noted that anomalous underscreening has been experimentally challenged,^19^ and despite considerable theoretical efforts^17,18,20−24^ there is at present no consensus as to its physical origin.

One often proposed explanation involves the role of clusters and their effect on the Debye screening length. For example, in a concentrated 1:1 electrolyte, the formation of neutral and/or weakly charged clusters in concentrated ionic solutions would act to reduce the number of independent charged species. If c* denotes the concentration of the *effective* screening charges (and we assume most clusters are either neutral or univalent), then the modified Debye length would be given by λ ≈ 1/c*^1/2^.^11,25^ Screening length measurements in the anomalous regime (high salt concentrations) have been fit to the relation λ ≈ *cl*_*B*_*d*^3^, where *l*_*B*_ is the Bjerrum length (*l*_*B*_ = β*e*_0_^2^/(4πε_0_ε_*r*_) and *d* is an average ionic diameter.^14^ Here,  is the inverse thermal temperature, *e*_0_ denotes the elementary charge, and ε_0_ is the permittivity of vacuum. Attributing underscreening to clustering and a modified Debye length would require c* ≈ 1/*c*^2^ in the anomalous region.

It is worthwhile to reflect upon the implications of such an explanation in a little more detail. Suppose we consider an electrolyte solution with a concentration well below the threshold value (∼1 M) above which underscreening occurs. Presumably, there will be some incipient clustering of ions at this concentration, but insufficient to affect the screening length significantly. Clustering at low concentration is driven by the electrostatic attractions between ions, but clusters remain finite due to the low chemical potential of the free ions (with which the aggregated ions remain in equilibrium). As the concentration increases, clustering is additionally aided by the lowering of the overall excluded volume due to ion aggregation. At concentrations lower than the threshold value for underscreening, addition of more ions will tend to lower the screening length, as the number of free ions at equilibrium will generally increase with the overall concentration. Let us now hypothesize that, once the concentration reaches the underscreening threshold value, the addition of more ions will cause the screening length to increase. Since we are now in the region of anomalous underscreening, this seems to suggest that effectively *all* the added ions will be aggregated to create *neutral* clusters. In addition, a portion of the original population of charged species (charged clusters and free ions) must also reorganize into neutral structures. If correct, this has the hallmarks of an apparent instability in the free energy of cluster formation. It suggests that, at a threshold concentration of free ions, clusters become unstable and experience accelerated growth, presumably because they have surpassed a critical size. This growth is arrested by a concurrent decrease in the free ion concentration. Experiments indicate that the concentration of effective screening charges would need to be some 10^4^ times lower than *c* in order to explain the upper levels of underscreening.^10−12,14,25^

In electrolyte theory, the Restricted Primitive Model (RPM) has been something of a “workhorse”. Here, ions are treated as charged hard spheres with diameter *d* and the solvent is simply modeled using a uniform dielectric constant ε_*r*_, which is generally greater than 1, due to the polarizability of solvent molecules. Thus, ion–ion interactions are described by ϕ_*ij*_(*r*), where
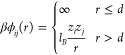
1Here, *z*_*i*_ and *z*_*j*_ denote the valencies of interacting ions *i* and *j*. Interestingly, hard sphere interactions tend to shorten the electrostatic screening length compared with λ_*D*_, due to interplay between hard sphere and electrostatic correlations.^16^

A clustering mechanism was recently explored using simulations of the RPM.^18^ The simulations showed that the propensity of clusters to form increases when the coupling strength and/or the electrolyte concentration increases and this does lead to underscreening. Furthermore, a clustering analysis was used to confirm that the measured screening length was consistent with using the apparent concentration of independent charge carriers, c* (charged clusters and free ions), i.e., λ ≈ 1/c*^1/2^. However, the underscreening observed was insufficient to explain experimental results. In particular, the simulations did not predict an increase in the observed screening length as the overall electrolyte concentration increases, i.e., the simulated c* *always increased* with *c*. It should be noted that these simulations also accounted for a reduction in ε_*r*_ with increasing *c*. Thus, the RPM is not able to reproduce the trends seen in experiments, which is perhaps not unanticipated, given the discussion above. That is, the RPM appears to have no inherent features that would suggest the possibility of anomalous clustering of the type discussed above.

Given this, it is interesting to consider other explanations to explain anomalous underscreening. In particular, we consider here the possibility that the decay length in experiments may be a reflection of the *size* of clusters, rather than their impact on the concentration of free charges. In the case of NaCl, the largest decay lengths measured in the anomalous regime are of the order of 30 Å.^12^ A pair of Na^+^ Cl^–^ ions in contact has a size of ∼6 Å, while water molecules have a diameter of ∼3 Å. Thus, one could envisage a small cluster of a few pairs of ions and hydrating water molecules to have a size of at least ∼30 Å. While such a mechanism has not been uncovered for the RPM, that may be a consequence of short-comings in that interaction model, which does not allow the formation of large enough clusters. Indeed, Safran and Pincus have presented scaling arguments that supports a strong relevance of cluster size.^26^

Most simple theories and simulation models of electrolytes do not explicitly include the solvent but allow it to be represented in a “primitive” fashion, via a relative dielectric constant, ε_*r*_, which is greater than unity due to the interaction of the ion charge with electrons and nuclear charges of the surrounding solvent molecules. Water is a polar solvent and can reorient and rearrange in response to the electric field of an ion. Experimentally, it is known that as the concentration of an aqueous electrolyte solution increases the overall dielectric constant decreases.^27−32^ This can be rationalized if we consider that hydrating water molecules are rotationally constrained by electric fields from the ions. As the salt concentration increases to molar levels, these constrained water molecules constitute an increasingly significant percentage of the solvent, which leads to a decreased overall dielectric response. This type of *dielectric saturation* has been the focus of some previous investigations at electrode surfaces, or in confined geometries.^28,30,33^ Theoretical investigations have explored this reduction in dielectric permittivity by correcting the mean field approach in a consistent manner,^21^ or else model it in terms of ion-specific effects.^34,35^ In protein small-angle X-ray scattering model fitting procedures, the formation of water hydration shells around charged (and uncharged) proteins, is a well-established phenomenon. A special treatment of hydration shells is needed, in order to produce a model fit that correctly describes the measured spectra.^36−38^

We propose to modify the RPM, based on physically plausible arguments. To maintain simplicity, we will explore a 2-body interaction model, but ultimately the effects we consider are best manifested using many-body forces. This notwithstanding, we consider it useful to understand the effect on screening lengths in the presence of much larger clusters than can be generated by the simple RPM. We use this model to roughly mimic NaCl and show that it saturates at a concentration close to its observed value, if not somewhat lower. While we by no means present this new model as an accurate representation of aqueous NaCl solutions, it does allow us to investigate screening behavior in electrolytes in the presence of much larger clusters than is generated by the RPM. This allows us to gain some further insight as to potential mechanisms that a cluster model can provide to explain anomalous underscreening apart from the usual assertion that it reduces the concentration of free charges.

A physically reasonable modification of the RPM, to account for dielectric saturation, would be to make ε_*r*_ in eq 1 a function of the average electrolyte concentration, as has been employed in previous RPM studies.^18^ This will increase the coupling strength between ions at higher average electrolyte concentration. However, even in dilute solutions, we expect that dielectric saturation will occur in regions where fluctuations cause a locally high concentration of ions. In such a region, solvent molecules will be displaced, and those that remain are subject to large electric fields.^30^ This suggests a spatial inhomogeneity in ε_*r*_ should occur as a result of changes in the ionic configurations. In principle, dielectric inhomogeneity should be modeled as a many-body phenomenon that qualitatively results in local changes to the electrostatic interactions. A collection of ions will produce large fields reducing the local dielectric constant and promoting clustering. Countering this will be an energetically unfavorable contribution due to the overlap of solvation shells of those ions. In other words, clustered ions are less well hydrated by water molecules and the total attractive water–ion interaction is thus expected to be weaker than for a fully dissociated solution. In this work, we consider a phenomenological manifestation of these mechanisms at the pairwise interaction level, in order to maintain computational simplicity.

In the model used here, ε_*r*_, is assumed to vary with the ionic separation, *r*. Specifically, it will have a reduced value when ions approach each other but has its *bulk* value when the ions are sufficiently separated. We will model ε_*r*_(*r*) as a linear ramp function of the following form:
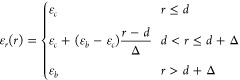
2with ε_*c*_ and ε_*b*_ denoting the *contact* and *bulk solvent* dielectric constant values, respectively. In general, we have ε_*c*_ < ε_*b*_ The slope of the linear ramp is defined by the parameter Δ, which is the thickness of the solvent layer and set to 3 Å. Consistent with this, we set ε_*b*_ = 78.3. The thus *modified* potential used here consists essentially of a short-ranged interaction (attractive between unlike ions, repulsive between like ions) in addition to a typical RPM interaction, wherein the latter assumes the uniform dielectric constant of the bulk solvent, ε_*b*_. While other implementations of the RPM have assumed a uniform dielectric constant, ε_*r*_, that decreases with electrolyte concentration to account dielectric saturation, we shall see below that a locally dependent ε_*r*_ of the type proposed here promotes more clustering, even compared to an RPM that uses ε_*r*_ = ε_*c*_ everywhere. We will use our potential model to explore both bulk simulations of the electrolyte as well as the solution in contact with charged surfaces.

We will only give a brief account of the simulation methods here, and refer to the Supporting Information (SI) for details. Canonical ensemble Metropolis Monte Carlo simulations were performed at 298 K for two geometries: a cubic simulation geometry, henceforth referred to as the *bulk*, and a parallelepiped geometry between two charged hard walls, referred to as the *slit*. Periodic boundary conditions were applied along (*x*, *y*, *z*) for the bulk simulations (with side-length of the cubic simulation box denoted as *L*) and along (*x*, *y*) for the slit simulations, with impenetrable charged hard walls situated at *z* = ±*H*/2. For slit systems, *L* denotes the length of the simulation parallelepiped along the (*x*, *y*)-axis, and *H* represents the slit width along the *z*-axis. The values of *L* and *H* are adjusted to accommodate the different electrolyte concentrations under investigation. For the bulk simulations, we employed Minimum Image (MI) truncation of the Coulomb interactions. The accuracy of this choice has been evaluated thoroughly in a recent work,^39^ by direct comparisons with more elaborate (and computationally expensive) Ewald summations. These comparisons verified that MI truncation leads to structurally accurate results for bulk systems. We have included yet another comparison, with the same conclusion, in the SI. For the slit systems, we employed the standard “charged sheet” method^40^ to account for long-ranged interactions. Notably, cluster moves were implemented for both systems, leading to crucial improvements of the statistical performance.

There are two free parameters in our model potential, which control the magnitude of the short-ranged attraction. We found that, at a high coupling strength (small *d* and ε_*c*_), the bulk solution appears to separate into an amorphous condensed phase in equilibrium with a dilute clustered phase. This could be viewed as the *saturation limit* for the solution, except in real electrolytes the condensed phase would be an ordered crystal. This phase instability is seen in Figure 1a, where we observe the response of the cation–cation pair correlation *g*_++_(*r*) to changes of ε_*c*_, at a concentration of 3.45 M. The development of a pronounced long-ranged slope in the tail of *g*_++_(*r*) is a signature of a phase separation. This was further supported by configurational snapshots in the SI, whereby visual inspection of the condensed phase suggests that it is noncrystalline in nature. While a more thorough structural analysis is lacking, we assert that determining the precise nature of the condensed phase is anyway not pertinent to the aims of this study. We can, however, plausibly argue that an amorphous condensed phase is not surprising, given the approximate nature of our model, wherein significant many-body effects are ignored. In particular, many-body effects are expected to promote clusters with ordered cores but labile outer regions, as dielectric saturation is larger toward the center of the clusters, while closer to the extremities solvent polarization remains large. On the other hand, the pairwise approximation, used in this study, somewhat erroneously strengthens ion–ion interactions *throughout* the cluster, as ameliorated by the choice of ε_*c*_. Thus, in order to predict a reasonable saturation concentration for the electrolyte model, a sensible choice for ε_*c*_ should fall between the bulk value of the solvent and the (small) internal dielectric constant of a crystal. However, such a choice will likely stabilize an amorphous condensed phase rather than a crystalline phase, given that ε_*c*_ will be larger than the expected dielectric response inside a crystal. Furthermore, the amorphous phase will also have less binding energy than that of the crystal, which suggests that our pairwise approximation will possibly predict less clustering as the solution concentration approaches saturation. KCl and NaCl have saturation concentrations of about 4.5–6 M, which suggests we should consider a similar saturation concentration in our modeling.

**Figure 1:**
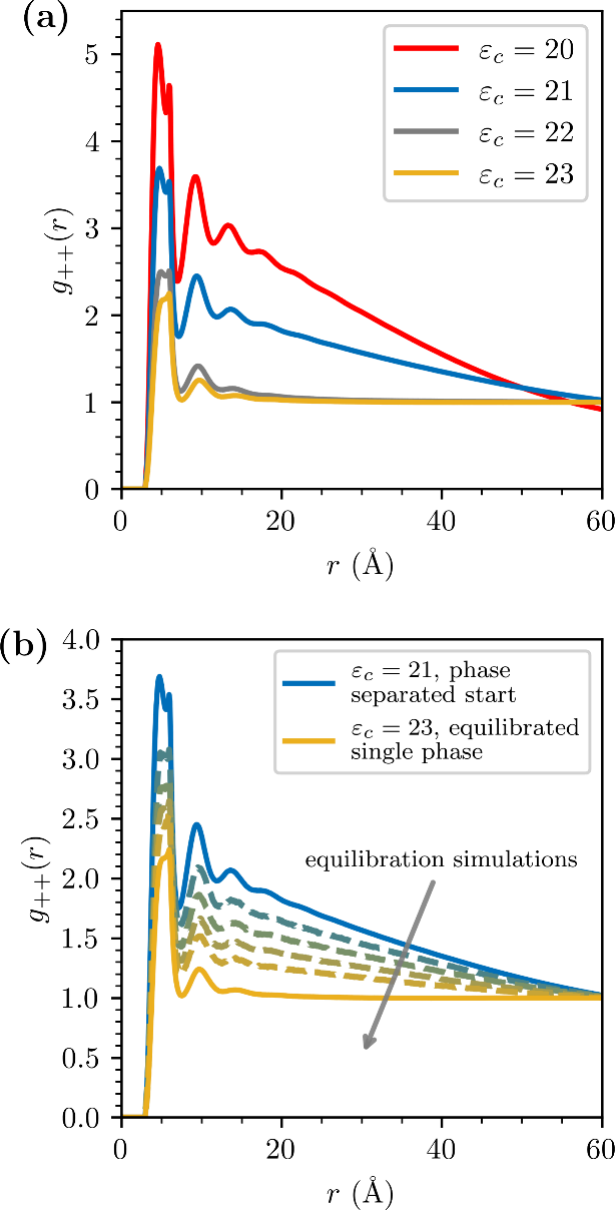
(a) Effect of changing the ε_*c*_ on the phase stability at 3.45 M. All systems contain 5000 ion pairs. (b) An illustration of the progression from a phase separated to homogenized system, for a system with *d* = 3 Å and ε_*c*_ = 23. The phase-separated system was created from simulations with ε_*c*_ = 21. Displayed are the cation–cation radial distribution functions from a set of short simulations (dashed lines), along with a sufficiently long final equilibrium simulation (with ε_*c*_ = 23).

Given that the aim of our study was primarily to explore the impact of clustering on electrostatic correlations, it was important that we select a value for ε_*c*_ so that the system displayed large but finite clusters. From Figures 1a and 1b, we see that, at 3.45 M, the system phase separates for ε_*c*_ values of 20 and 21, but not when ε_*c*_ is above 22 (for *d* = 3 Å). Thus, we have set ε_*c*_ = 23 for all subsequent simulations. This value provides a significant degree of clustering in our model while the solution remains unsaturated at least up to 3.45 M. A different choice of *d* would lead to a different choice of ε_*c*_, as described in the SI.

It is of course possible that the system is *metastable* rather than stable for ε_*c*_ = 23 at 3.45 M. However, we have made tests ensuring that a metastable scenario is highly unlikely. Specifically, we have (several times) initiated simulations from a phase-separated system, at ε_*c*_ = 21, only to find that, upon switching to ε_*c*_ = 23, the system transitions to a single phase state at equilibrium. This is illustrated in Figure 1b, where we depict how the pronounced long-ranged gradient of the initial radial distribution function (indicating a phase-separated system), gradually disappears.

We reiterate that our potential model introduces an additional short-ranged asymmetric interaction, which promotes association between unlike ions and the formation of large clusters. This cluster formation, as a function of the electrolyte concentration, can be readily observed by analyzing radial distribution functions, *g*_*ij*_(*r*), between ions of valency *i* and *j* (i.e., *i*, *j* = ± ), as illustrated in Figures 2a and 2b). By symmetry, *g*_– –_(*r*) = *g*_+ +_(*r*) and *g*_+ –_(*r*) = *g*_– +_(*r*). Thus, it is prudent to use (*g*_+ +_(*r*) + *g*_– –_(*r*))/2 and (*g*_+ –_(*r*) + *g*_– +_(*r*))/2 for the like and unlike correlation functions. We can define the total density correlation function *g*_*nn*_(*r*) = (*g*_+ +_(*r*) + *g*_– –_(*r*) + *g*_+ –_(*r*) + *g*_– +_(*r*))/4 (the particle density correlation around any ion), as well as the so-called “charge–charge correlation function” *g*_*cc*_(*r*) = (*g*_+ –_(*r*) + *g*_– +_(*r*) – *g*_+ +_(*r*) – *g*_– –_(*r*))/4 (the counter-charge density around an ion). The charge–charge correlation functions are shown in Figure 2c. Using linear response theory, it is possible to relate the interaction free energy between two charged surfaces (at large separation) to these correlation functions at long range. As charged surfaces perturb both the charge and particle densities of the electrolyte contained between them, the interaction between the surfaces at large separations is dictated either by *g*_*cc*_(*r*) or *g*_*nn*_(*r*), whichever has the longest range. Classical mean-field theory, wherein ions are assumed to respond only to the mean electrostatic potential, asserts that *h*_*cc*_(*r*) has a Yukawa form, *h*_*cc*_(*r*) ≈ exp(−*r*/λ_*D*_)/*r* and *h*_*nn*_(*r*) is much shorter-ranged (*h*_αβ_(*r*) = *g*(*r*)_αβ_ – 1). In fact, linearized mean-field analysis predicts that the charged surfaces will not alter the total ionic density between them at all, *h*_*nn*_(*r*) = 0, whereas, to second order, we have *h*_*nn*_(*r*) ≈ exp(−2*r*/λ_*D*_)/*r*^2^. This qualitative picture may change in the presence of strong correlations and, as in our case, with the formation of large clusters.

**Figure 2:**
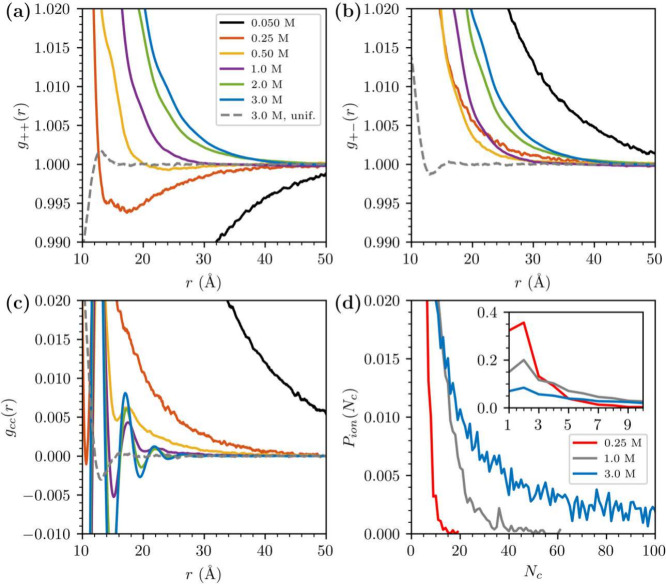
Results from bulk simulations. (a) Cation–cation (or anion–anion) resolved radial distribution functions, *g*_+ +_(*r*) (on average identical to *g*_– –_(*r*)), for the local dielectric saturation model (full line), and a uniform low-dielectric constant (a uniform value of ε_*r*_ = 23) model (dashed line). (In order to improve statistics, we have, in reality, measured “*g*_+ +_(*r*)” as (*g*_+ +_(*r*) + *g*_– –_(*r*))/2) (b) Cation–anion resolved radial distribution functions, *g*_+ –_(*r*). (c) *g*_*cc*_(*r*). (d) Probability of an ion to be part of a cluster of size *N*_*c*_, *P*_ion_(*N*_*c*_), at three different concentrations. An ion must be within a distance δ or less, of at least one other ion within a cluster, in order to be a member of that cluster. We have set δ = *d* + 0.5 Å. The inset is a focus on small clusters.

At the lowest concentration investigated (0.05 M), we observe what could be described as *mean-field* behavior for the correlation functions. Here, *g*_+ +_(*r*) displays a co-ion exclusion region, while *g*_+ –_(*r*) illustrates counterion attraction, so that *h*_+ +_(*r*) ≈ −*h*_+ –_(*r*) as they approach zero. Between 0.25 and 0.5 M a peak appears in *g*_+ +_(*r*) at short-range, which then displays co-ion exclusion at larger distances. As the concentration increases, however, the co-ion exclusion region diminishes in size so that at 0.5 M it has almost vanished. On the other hand, *g*_+ –_(*r*) still displays an attraction between counterions, but its range appears to decrease, which is actually qualitatively consistent with the mean-field behavior. In Figure 2c, we observe the a reduction in range of *g*_*cc*_(*r*) as well. In addition, oscillations appear at short-range at 0.5 M, which, together with the behavior of *g*_+ +_(*r*), suggests accumulation of ions into clusters. However, even in this concentration range, the *tails* of these correlation functions are still Yukawa like and, as we show below (and in the SI), asymptotic fitting gives a correlation length which is greater than λ_*D*_, indicating some degree of underscreening.

At even higher concentrations (1 and 3 M), we note the complete disappearance of the co-ion exclusion region in *g*_+ +_(*r*). This is accompanied by apparent coincidence of *g*_+ +_(*r*) and *g*_+ –_(*r*) at longer range, as indicated by the diminished range of their difference, *g*_*cc*_(*r*), as shown in Figure 2c. That is, at lower concentrations, the long-range decays of all these correlation functions were similar, albeit *g*_+ +_(*r*) and *g*_+ –_(*r*) approach unity from below and above, respectively. Indeed, in the SI, we show that they can be all reasonably well-fitted to a Yukawa form, ∼exp(−*r*/λ)/*r*, where λ is generally larger than the Debye length. However, at 1 and 3 M, *g*_+ +_(*r*) and *g*_+–_(*r*) both approach unity from above and, their range is significantly longer than *g*_*cc*_(*r*). These results indicate that there is a great deal of clustering occurring, causing much longer range correlations in the density, *g*_*nn*_(*r*), compared with the charge, *g*_*cc*_(*r*). The correlation length of *g*_*nn*_(*r*) will be determined by the typical cluster size. In Figure 2d, we show the probability, *P*_ion_(*N*_*c*_), that an ion is a member of a cluster of size *N*_*c*_ for different concentrations, and the growth in cluster sizes is apparent as the ion concentration increases. The growth in clusters is accompanied by more rapid screening of charges. This effect on charge screening is also qualitatively predicted by mean-field theories, as the Debye length decreases with concentration. But here, we also see that *g*_*cc*_(*r*) loses its Yukawa form and becomes oscillatory, so hard-core correlations within dense clusters are clearly playing a role. Most clusters are expected to be close to neutral, which is why the difference between cation–cation and cation–anion correlations at long-range vanishes. What has not been previously reported in other theoretical treatments of electrolyte models (as far as we are aware), is the significant *increase* in range of *g*_*nn*_(*r*) as a function of concentration, compared with *g*_*cc*_(*r*), see Supporting Information (SI).

Thus, our model predicts that, at a concentration of ∼1 M, the interaction between charged surfaces will begin to be dominated by density correlations, as measured by *g*_*nn*_(*r*), rather than charge correlations (from *g*_*cc*_(*r*)), which were significant at lower concentrations. In our model, density correlations are affected by cluster formation and their typical size, whereas charge correlations are determined by the availability of screening charges.

We argue that our modification of the RPM gives rise to clusters that are much larger than usual applications of the RPM. To illustrate this, we also calculated the correlation functions at 3 M for a simple RPM case, where the uniform dielectric constant was chosen to be ε_*r*_ = ε_*c*_ = 23. That is, we assumed dielectric saturation occurs over the *full range* of the Coulomb interaction, rather than just close to ion–ion contact. We see (Figure 2a) that *g*_+ +_(*r*) then displays a short-ranged co-ion exclusion region superimposed on an oscillatory profile, with no long-ranged tail as observed in our modified RPM. Similarly, *g*_+ –_(*r*) shows a short-ranged counterion enhancement with oscillations, again without a long-ranged tail (Figure 2b. The corresponding *g*_*cc*_(*r*) is also oscillatory, but with amplitude much smaller than the modified RPM (Figure 2c), which is evidence of much larger clustering induced by the modified RPM. It is clear that the additional short-ranged interaction introduced in our modified RPM reduces the incentive for ions to dissociate from clusters once they are formed. If dielectric saturation is assumed to occur everywhere, even in regions of low ionic density, there is less of an advantage for ions to cluster. Note that this affect is ameliorated by the decreased ion solvation expected to occur in clusters, an effect which must be accounted for when choosing a suitable value for ε_*c*_.

It may seem surprising that the *range* of a reduced dielectric response has such a strong impact on the cluster forming tendency. However, one should be cognizant of the fact that, with a uniform and low dielectric constant, there are inevitable strong repulsions between like charges in a cluster. These repulsions are considerably weaker if the reduced dielectric response is local, mainly influencing the interaction between charges of opposite sign.

In previous work, we developed a simulation method that uses a modified Widom technique to estimate the long-range decay length of *g*_*cc*_(*r*), which is very accurate if one can assume a Yukawa form, *g*_*cc*_(*r*) ≈ 1 + *A* exp(−*r*/λ)/*r*.^39^ The method is briefly described in the SI. The results for the correlation functions calculated above are compared with the standard Debye screening length (λ_*D*_) in Figure 3. Note that, while the Yukawa form is only really valid below 1 M, our method is nevertheless still able to extract an *effective* screening length at the higher concentrations. This is because it is based on a linear expansion of the free-energy functional that predicts a screening length from an ensemble average. This average is still calculable even in cases where assumptions which lead to a Yukawa form breakdown. In Figure 3, we do observe some underscreening, the degree of which is much smaller than that suggested by SFA experiments.^10−14^ In particular, we do not observe an increase in screening length with concentration. In any case, as described above, the decay length of *g*_*cc*_(*r*) is not the dominant one above 1 M, but rather that of *g*_*nn*_(*r*). Interestingly, by the use of asymptotic analysis techniques, researchers have previously determined that such a scenario is theoretically possible.^16,41^ That is, even in the RPM, a transition may occur from charge–charge correlation to a density–density dominated correlation in the asymptotic (long-range) regime.

**Figure 3:**
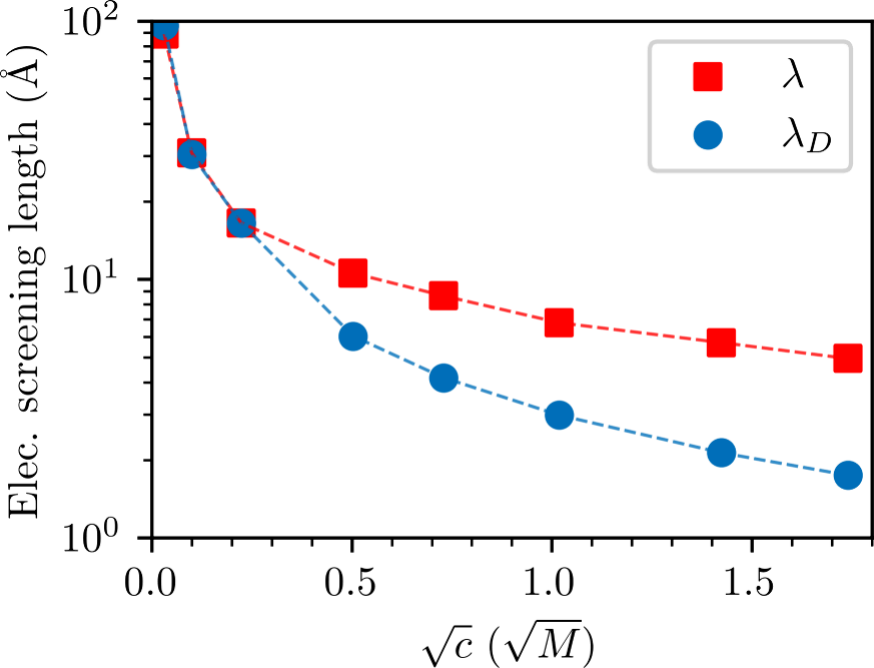
Effective electrostatic screening lengths obtained via the modified Widom technique, and the corresponding Debye screening lengths. The dashed lines serve as a guide to the eye.

Finally, we turn our attention to structural properties of our modified RPM, in the presence of two macroscopic flat and negatively charged surfaces. For these systems, we have chosen a simulation model with a slit geometry. The canonical slit simulations enable us to study the behavior of the model in a system that approximates the experimental SFA setup: an electrolyte confined between two macroscopic charged surfaces. We investigated three concentrations at a constant inverse surface charge density of −70 Å^2^/e. The resulting ion density distributions, *n*_+_(*z*) and *n*_–_(*z*), where *z* is the direction normal to the surfaces, are presented in Figure 4a. We can observe a significant difference between *n*_+_(*z*) and *n*_–_(*z*) at the midplane between the surfaces, even when these are 50 Å apart. This is in stark contrast to mean-field predictions, since the midplane is more than approximately 13 Debye lengths distant from the surfaces, at the highest investigated bulk concentration (∼2.6 M). In order to clarify our results further, we plot the density difference, Δ*n*(*z*) ≡ *n*_+_(*z*) – *n*_–_(*z*), in Figure 4b. Even though the overall Δ*n*(*z*) profile does drop as the salt concentration increases, we note that the salt dependence is quite weak, and that a significant long-ranged tail persists also for very high ionic strengths. The scenario would again be quite different with a model using a uniform dielectric constant (even ε_*r*_ = 23), in which case Δ*n*(*z*) rapidly vanishes away from a charged surface, at high concentrations. This is explicitly shown by the dashed line in Figure 4b. While we have not measured the net interaction between such charged surfaces in this study, the long-ranged tail of Δ*n*(*z*) clearly implies a slowly decaying surface force. This force will be quantified in future simulation work.

**Figure 4:**
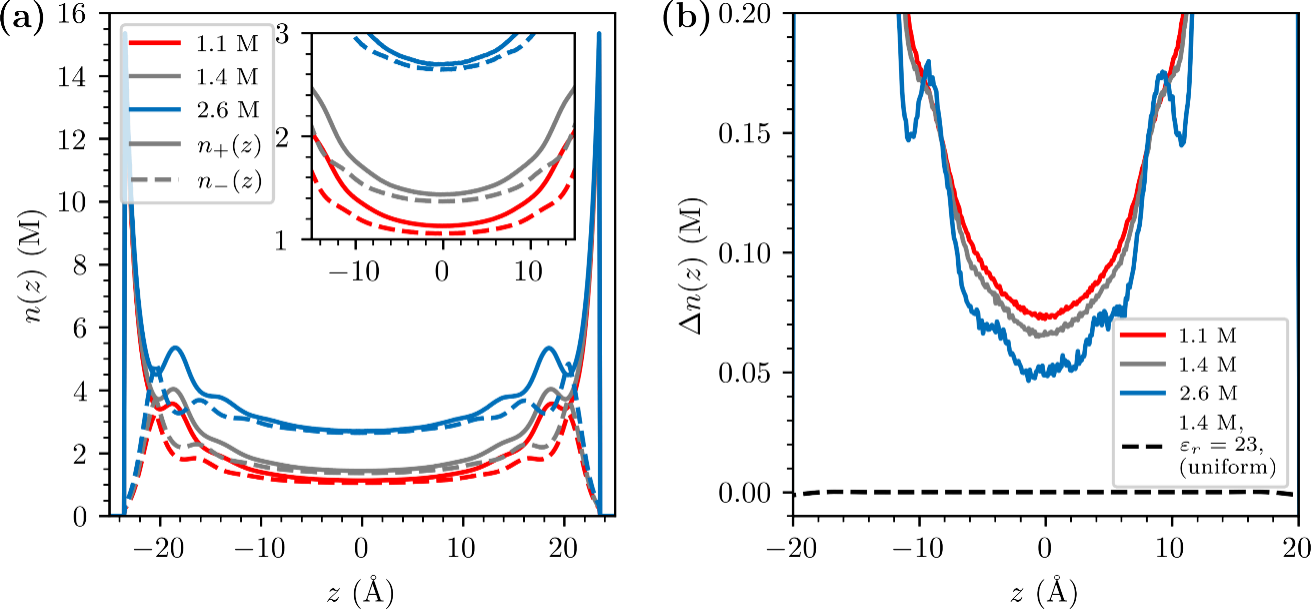
Results from the slit geometry simulations, with negatively charged surfaces. (a) Concentrations of cations (solid lines) and anions (dashed lines) along the *z*-axis, for three different simulated bulk concentrations (these are estimated from mid plane values). (b) The concentration difference between cations and anions along the *z*-axis. Here, we have also added data from RPM simulations with a uniform dielectric constant of 23 at 1.4 M (dashed line).

Considering the fact that experimental approaches extract the effective correlation lengths,^14^ which may or may not be purely a result of charge–charge correlations, the observation of a transition from the expected charge–charge asymptotic domination to a density–density domination at high concentrations strongly supports the hypothesis that cluster formation, has a crucial influence on anomalous underscreening effects. It should be noted that Safran and Pincus have recently presented scaling arguments according to which the screening length is expected to depend on the cluster size.^26^

This theoretical study has investigated the influence of local dielectric saturation on the structure of electrolytes modeled by the RPM. We demonstrate that local dielectric saturation induces significant clustering. Interactions between such clusters dominate the asymptotic correlations for systems approaching or exceeding concentrations of about 1 M. When charged surfaces are immersed in such solutions, a net charge density develops, that decays quite slowly with the transverse distance to these surfaces. This suggests that there might be long-ranged interactions between such surfaces, commensurate with observations by SFA.

## Data Availability

All codes used for simulations, along with the data generated, is freely available, via this Github repository: https://github.com/janneforsman/local_dielectric_saturation.

## Supporting Information Available

Detailed simulation methods, and further analyses (PDF)

## References

[ref1] Israelachvili J. N. Intermolecular and Surface Forces, 2nd Ed.; Academic Press: London, 1991.

[ref2] Evans F. A.; Wennerström H. The Colloidal Domain: Where Physics, Chemistry, Biology and Technology Meet; VCH Publishers: New York, 1994.

[ref3] Holm C.; Kekicheff P.; Podgornik R. Electrostatic Effects in Soft Matter and Biophysics; Kluwer Academic Publishers: Dordrecht, The Netherlands, 2001.

[ref4] Derjaguin B. V.; Landau L. Theory of the Stability of Strongly Charged Lyophobic Sols and of the Adhesion of Strongly Charged Particles in Solutions of Electrolytes. Acta Phys. Chim. URSS 1941, 14, 633–662.

[ref5] Verwey E. J. W.; Overbeek J. T. G. Theory of the Stability of Lyophobic Colloids; Elsevier Publishing Company, Inc.: Amsterdam, 1948.

[ref6] Nordholm S. Generalized van der Waals theory. XII. Application to ionic solutions. Aust. J. Chem. 1984, 37, 1 10.1071/CH9840001.

[ref7] Guldbrand L.; Jönsson B.; Wennerström H.; Linse P. Electrical Double Layer Forces, A Monte Carlo Study. J. Chem. Phys. 1984, 80, 2221 10.1063/1.446912.

[ref8] Kjellander R.; Marcelja S. Interaction of charged surfaces in electrolyte solutions. Chem. Phys. Lett. 1986, 127, 402–407. 10.1016/0009-2614(86)80304-9.

[ref9] Valleau J.; Ivkov R.; Torrie G. M. J. Phys. Chem. 1991, 95, 520 10.1063/1.461452.

[ref10] Gebbie M. A.; Valtiner M.; Banquy X.; Fox E. T.; Henderson W. A.; Israelachvili J. N. Ionic liquids behave as dilute electrolyte solutions. Proc. Natl. Acad. Sci. U.S.A. 2013, 110, 9674–9679. 10.1073/pnas.1307871110. 23716690 PMC3683794

[ref11] Gebbie M. A.; Dobbs H. A.; Valtiner M.; Israelachvili J. N. Long-range electrostatic screening in ionic liquids. Proc. Natl. Acad. Sci. U.S.A. 2015, 112, 7432–7437. 10.1073/pnas.1508366112. 26040001 PMC4475974

[ref12] Smith A. M.; Lee A. A.; Perkin S. The Electrostatic Screening Length in Concentrated Electrolytes Increases with Concentration. J. Phys. Chem. Lett. 2016, 7, 2157–2163. 10.1021/acs.jpclett.6b00867. 27216986

[ref13] Fung Y. K. C.; Perkin S. Structure and anomalous underscreening in ethylammonium nitrate solutions confined between two mica surfaces. Faraday Discuss. 2023, 246, 370–386. 10.1039/D3FD00042G. 37458200 PMC10568257

[ref14] Lee A. A.; Perez-Martinez C. S.; Smith A. M.; Perkin S. Underscreening in concentrated electrolytes. Faraday Discuss. 2017, 199, 239–259. 10.1039/C6FD00250A. 28466925

[ref15] Yuan H.; Deng W.; Zhu X.; Liu G.; Craig V. S. J. Colloidal Systems in Concentrated Electrolyte Solutions Exhibit Re-entrant Long-Range Electrostatic Interactions due to Underscreening. Langmuir 2022, 38, 6164–6173. 10.1021/acs.langmuir.2c00519. 35512818 PMC9119301

[ref16] Attard P. Asymptotic analysis of primitive model electrolytes and the electrical double layer. Phys. Rev. E 1993, 48, 3604–3621. 10.1103/PhysRevE.48.3604. 9961018

[ref17] Coupette F.; Lee A. A.; Härtel A. Screening Lengths in Ionic Fluids. Phys. Rev. Lett. 2018, 121, 075501 10.1103/PhysRevLett.121.075501. 30169089

[ref18] Härtel A.; Bültmann M.; Coupette F. Anomalous Underscreening in the Restricted Primitive Model. Phys. Rev. Lett. 2023, 130, 108202 10.1103/PhysRevLett.130.108202. 36962045

[ref19] Kumar S.; Cats P.; Alotaibi M. B.; Ayirala S. C.; Yousef A. A.; van Roij R.; Siretanu I.; Mugele F. Absence of anomalous underscreening in highly concentrated aqueous electrolytes confined between smooth silica surfaces. J. Colloid Interface Sci. 2022, 622, 819–827. 10.1016/j.jcis.2022.05.004. 35561602

[ref20] Rotenberg B.; Bernard O.; Hansen J.-P. Underscreening in ionic liquids: a first principles analysis. J. Phys.: Condens. Matter 2018, 30, 054005 10.1088/1361-648X/aaa3ac. 29271363

[ref21] Kjellander R. A multiple decay-length extension of the Debye–Hückel theory: to achieve high accuracy also for concentrated solutions and explain under-screening in dilute symmetric electrolytes. Phys. Chem. Chem. Phys. 2020, 22, 23952–23985. 10.1039/D0CP02742A. 33073810

[ref22] Coles S. W.; Park C.; Nikam R.; Kanduc M.; Dzubiella J.; Rotenberg B. Correlation Length in Concentrated Electrolytes: Insights from All-Atom Molecular Dynamics Simulations. J. Phys. Chem. B 2020, 124, 1778–1786. 10.1021/acs.jpcb.9b10542. 32031810

[ref23] Zeman J.; Kondrat S.; Holm C. Bulk ionic screening lengths from extremely large-scale molecular dynamics simulations. Chem. Commun. 2020, 56, 15635–15638. 10.1039/D0CC05023G. 33283802

[ref24] Cats P.; Evans R.; Härtel A.; van Roij R. Primitive model electrolytes in the near and far field: Decay lengths from DFT and simulations. J. Chem. Phys. 2021, 154, 124504 10.1063/5.0039619. 33810662

[ref25] Ma K.; Forsman J.; Woodward C. E. Influence of ion pairing in ionic liquids on electrical double layer structures and surface force using classical density functional approach. J. Chem. Phys. 2015, 142, 174704 10.1063/1.4919314. 25956113

[ref26] Safran S. A.; Pincus P. A. Scaling perspectives of underscreening in concentrated electrolyte solutions. Soft Matter 2023, 19, 7907–7911. 10.1039/D3SM01094E. 37823228

[ref27] Hasted J. B.; Ritson D. M.; Collie C. H. Dielectric Properties of Aqueous Ionic Solutions. Parts I and II. J. Chem. Phys. 1948, 16, 1–21. 10.1063/1.1746645.

[ref28] de Souza J.; Kornyshev A. A.; Bazant M. Z. Polar liquids at charged interfaces: A dipolar shell theory. J. Chem. Phys. 2022, 156, 244705 10.1063/5.0096439. 35778078

[ref29] Conway B.; Marshall S. Some common problems concerning solvent polarization and dielectric behaviour at ions and electrode interfaces. Aust. J. Chem. 1983, 36, 2145–2161. 10.1071/CH9832145.

[ref30] Bonthuis D. J.; Gekle S.; Netz R. R. Profile of the Static Permittivity Tensor of Water at Interfaces: Consequences for Capacitance, Hydration Interaction and Ion Adsorption. Langmuir 2012, 28, 7679–7694. 10.1021/la2051564. 22414296

[ref31] Danielewicz-Ferchmin I.; Banachowicz E.; Ferchmin A. Dielectric saturation in water as quantitative measure of formation of well-defined hydration shells of ions at various temperatures and pressures. Vapor–liquid equilibrium case. J. Mol. Liq. 2013, 187, 157–164. 10.1016/j.molliq.2013.06.005.

[ref32] Adar R. M.; Markovich T.; Levy A.; Orland H.; Andelman D. Dielectric constant of ionic solutions: Combined effects of correlations and excluded volume. J. Chem. Phys. 2018, 149, 054504 10.1063/1.5042235. 30089391

[ref33] Underwood T. R.; Bourg I. C. Dielectric Properties of Water in Charged Nanopores. J. Phys. Chem. B 2022, 126, 2688–2698. 10.1021/acs.jpcb.1c09688. 35362980 PMC10114093

[ref34] Ben-Yaakov D.; Andelman D.; Podgornik R. Dielectric decrement as a source of ion-specific effects. J. Chem. Phys. 2011, 134, 074705 10.1063/1.3549915. 21341867

[ref35] Hubbard J. B.; Colonomos P.; Wolynes P. G. Molecular theory of solvated ion dynamics. III. The kinetic dielectric decrement. J. Chem. Phys. 1979, 71, 2652–2661. 10.1063/1.438622.

[ref36] Knight C. J.; Hub J. S. WAXSiS: a web server for the calculation of SAXS/WAXS curves based on explicit-solvent molecular dynamics. Nucleic Acids Res. 2015, 43, W225–W230. 10.1093/nar/gkv309. 25855813 PMC4489308

[ref37] Hansen J.; Uthayakumar R.; Pedersen J. S.; Egelhaaf S. U.; Platten F. Interactions in protein solutions close to liquid–liquid phase separation: ethanol reduces attractions via changes of the dielectric solution properties. Phys. Chem. Chem. Phys. 2021, 23, 22384–22394. 10.1039/D1CP03210K. 34608908

[ref38] Hansen J.; Pedersen J. N.; Pedersen J. S.; Egelhaaf S. U.; Platten F. Universal effective interactions of globular proteins close to liquid–liquid phase separation: Corresponding-states behavior reflected in the structure factor. J. Chem. Phys. 2022, 156, 244903 10.1063/5.0088601. 35778071

[ref39] Forsman J.; Ribar D.; Woodward C. E. An efficient method to establish electrostatic screening lengths of restricted primitive model electrolytes. Phys. Chem. Chem. Phys. 2024, 26, 19921–19933. 10.1039/D4CP00546E. 38990567

[ref40] Torrie G. M.; Valleau J. P. Electrical double layers. I. Monte Carlo study of a uniformly charged surface. J. Chem. Phys. 1980, 73, 5807–5816. 10.1063/1.440065.

[ref41] Leote de Carvalho R.; Evans R. The decay of correlations in ionic fluids. Mol. Phys. 1994, 83, 619–654. 10.1080/00268979400101491.

