# Supplementary material for: Cluster Formation Induced by Local Dielectric Saturation in Restricted Primitive Model Electrolytes
Source: J Phys Chem Lett. 2024 Aug 7;15(32):8326–33. doi: 10.1021/acs.jpclett.4c01829 (PMC11331514; doi:10.1021/acs.jpclett.4c01829)
Supplement: Supplementary file 1 — jz4c01829_si_001.pdf [file jz4c01829_si_001.pdf]

Supporting information for:

Cluster Formation induced by local dielectric saturation in Restricted Primitive Model Electrolytes

David Ribar[a], Clifford E. Woodward[b], Sture Nordholm[c], and Jan Forsman[a,*]

[a] *Computational Chemistry, Lund University, P.O.Box 124, S-221 00 Lund, Sweden*

[b] *School of Physical, Environmental and Mathematical Sciences University College, University of New South Wales, ADFA Canberra ACT 2600, Australia*

[c] *Department of Chemistry and Molecular Biology, The University of Gothenburg, 412 96 Gothenburg, Sweden*

*Corresponding author: jan.forsman@compchem.lu.se

(Dated: 2024-08-02)

S1 Simulation details

All simulations were performed in the canonical ensemble at $T = 298$ K, with the Metropolis Monte Carlo method, and with a particle hard-sphere diameter of $d = 3$ Å, unless otherwise specified. The minimum image (MI) truncation was used for the interaction energy calculations, which was shown to be satisfactory for an RPM type system by Forsman *et al.* [1], where a comparison revealed that the MI truncation produces essentially identical results for RPM systems, to more elaborate long-ranged interaction management methods. For completeness, we have made a similar comparison with our modified RPM, in Figure 1. The agreement is quite satisfactory. The MI option is computationally cheaper, and

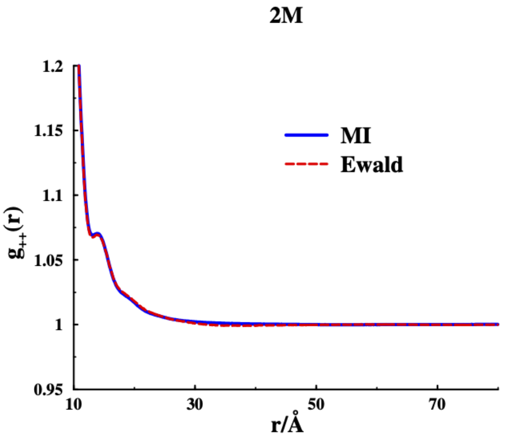

Figure 1: Structural comparison between MI (solid) and Ewald (dashed) based simulations, at about 2M.

therefore our preferred method. After system equilibration was achieved, we sampled the ion resolved radial distribution functions $g_{++}(r), g_{+-}(r)$ and $g_{--}(r)$. We simulated the bulk electrolyte in a cubic simulation box, defined by L. Periodic boundary conditions were applied along all coordinate axes.

For the slit geometry we utilised a simulation parallelepiped, where $L = 172$ Å defines the extension along the x and y directions, and $H = 50$ Å denotes the slit height in the z direction, with impenetrable (hard) walls at $\pm H/2$. Periodic boundary conditions were applied along the (x, y) coordinate axes. 1500 anions and 2348 cations were used, resulting in an inverse surface charge density $1/\sigma = -70\text{Å}^2/e$. To

account for the finite size of the simulation parallelepiped with the MI truncation, we implemented a mean field approach utilising an external potential of smeared out charge densities parallel to the slit [2, 3] via

$$\beta\phi^{ext}(z) = \frac{l_B}{e}\int_0^H < \Delta n(z') > (-2\pi|z-z'| - \psi^{ext}(|z-z'|))dz' \tag{1}$$

with $< \Delta n(z) >=< n_+(z) - n_-(z) >$ denoting the average net charge density, as obtained from a previous equilibrium simulation. $\psi^{ext}(z)$ is a "charged sheet" mean-field contribution from the square simulation box region of such a sheet at z. This "internal" contribution should be subtracted from the infinite surface contribution, as shown in eq. (1), in order to provide the net "external" (mean-field) interaction. $\psi^{ext}(z)$ is given by:

$$\psi^{ext}(z) = 4L\ln\left[\frac{\sqrt{\frac{L^2}{2}+z^2}}{\sqrt{\frac{L^2}{4}+z^2}}\right] - 2z\arcsin\left[\frac{\frac{L^4}{16}-z^4-\frac{L^2z^2}{2}}{\frac{L^2}{4}+z^2}+\frac{\pi}{2}\right] \tag{2}$$

The interaction energy, $\beta U_i^{ext}(z)$ of an ion i, of valency z_i, with this mean-field representation of charges outside of the simulation box is thus $\beta U_i^{ext}(z) = z_i e\beta\phi^{ext}(z)$.

The external potential is computed at the start of the simulation for discrete z distances, utilising the average net charge density from a previous simulation, and is linearly interpolated to the actual particle z coordinate value for the corresponding energy calculations. Naturally, for the slit geometry, the radial distribution functions were replaced with calculating the ion number densities along the z-axis.

S2 Calculating the electrostatic screening length, λ

Here we give a brief account for the modified Widom method to calculate the effective electrostatic screening length, λ. A more detailed account is provided in a recent publication [1]. In the presence of an average electrostatic potential $\psi(\mathbf{r}_1)$, we can express the density $n_i(\mathbf{r}_1)$ of ion species i at $\mathbf{r}_1$ as:

$$n_i(\mathbf{r}_1) = n_b e^{-\beta\left(z_i e\psi(\mathbf{r}_1)+\frac{\delta F_{ex}}{\delta n_i(\mathbf{r}_1)}-\mu_i^{ex}\right)} \tag{3}$$

We define the excess chemical potential of species i, μ_i^{ex}:

$$\mu_i^{ex} = \frac{\delta F_{ex}}{\delta n_i}(bulk) \tag{4}$$

where F_{ex} is the excess free energy. The excess direct correlation function, c_{ij}, is given by:

$$\beta\frac{\delta^2 F_{ex}}{\delta n_i(\mathbf{r}_1)\delta n_j(\mathbf{r}_2)}(bulk) = -c_{ij}(|\mathbf{r}_1-\mathbf{r}_2|) \tag{5}$$

and the net charge density, ρ:

$$\rho(\mathbf{r}_1) = e\sum_i z_i n_i(\mathbf{r}_1) \tag{6}$$

Defining $\Delta n_i \equiv n_i - n_b$, where n_b is the bulk salt density we get, upon linearisation of eq.(3):

$$\rho(\mathbf{r}_1) = -\psi(\mathbf{r}_1)/\lambda_D^2 + n_b e\sum_i\sum_j\int z_i\Delta n_j(\mathbf{r}_2)c_{ij}^{ex}(|\mathbf{r}_1-\mathbf{r}_2|)d\mathbf{r}_2 \tag{7}$$

where we have utilised the standard expression for the inverse Debye screening length, λ_D:

$$\lambda_D^{-2} = \beta n_b\sum_i(ez_i)^2 \tag{8}$$

By utilising the symmetry of an RPM electrolyte, eq.(7) can be further simplified:

$$\rho(\mathbf{r}_1) = -\psi(\mathbf{r}_1)/\lambda_D^2 + n_b\int\rho(\mathbf{r}_2)(c_{++}^{ex}(|\mathbf{r}_1-\mathbf{r}_2|) - c_{+-}^{ex}(|\mathbf{r}_1-\mathbf{r}_2|))d\mathbf{r}_2 \tag{9}$$

with an obvious notation. We now make the assumption that for a slow asymptotic decay, we have:

$$\rho(\mathbf{r}_1) \approx -\psi(\mathbf{r}_1)/\lambda_D^2 + \rho(\mathbf{r}_1)n_b\int\left(c_{++}^{ex}(|\mathbf{r}_1-\mathbf{r}_2|) - c_{+-}^{ex}(|\mathbf{r}_1-\mathbf{r}_2|)\right)d\mathbf{r}_2 \tag{10}$$

which finally leads to:

$$\rho(\mathbf{r}_1) = -\frac{\psi(\mathbf{r}_1)}{\alpha\lambda_D^2} = -\frac{\psi(\mathbf{r}_1)}{\lambda^2} \tag{11}$$

where we have defined

$$\alpha = 1 - n_b \int \left(c_{++}^{ex}(|\mathbf{r}_1 - \mathbf{r}_2|) - c_{+-}^{ex}(|\mathbf{r}_1 - \mathbf{r}_2|) \right) d\mathbf{r}_2 \tag{12}$$

from which the final electrostatic screening length, $\lambda = \lambda_D\sqrt{\alpha}$, is established. Noting that

$$\beta \frac{\partial \mu_i^{ex}}{\partial n_j} = -\int c_{ij}^{ex}(|\mathbf{r}_1 - \mathbf{r}_2|) d\mathbf{r}_2 \tag{13}$$

we can rewrite the screening correction factor α as:

$$\alpha = 1 + \beta n_b \left(\frac{\partial \mu_+^{ex}}{\partial n_+} - \frac{\partial \mu_+^{ex}}{\partial n_-} \right) \tag{14}$$

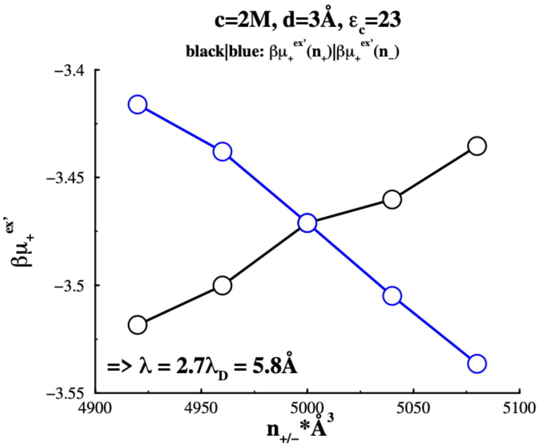

Figure 2: Simulated values of $\mu_+^{ex'}$ and $\mu_-^{ex'}$ at a salt concentration of about 2M, using the inverse Widom method, as described in the main text. The lines are a guide to the eye.

The excess chemical potentials of separate ionic species in the bulk were estimated using the inverse Widom technique [4] using the Sloth and Sörensen [5] electroneutrality correction. Strictly speaking, the inverse Widom method will not sample the excluded volume hard-sphere part, $\mu^{ex}(HS)$, of the ion chemical potentials. However, due to the size symmetry of the RPM, this will not give any net contribution to the difference in eq.(14). In order to simplify the notation, we therefore define $\mu_+^{ex'} \equiv \mu_+^{ex} - \mu_+^{HS}$ where μ_+^{HS} is the hard-sphere contribution to the chemical potential. Figure 2 displays resulting excess chemical potential data for a 5000 ion pair system, with $L = 160$ Å, leading to a concentration of about 2 M (2.027 M to be more precise). The derivatives have in this case been estimated from linear fits to slopes of $\mu_+^{ex'}$ and $\mu_-^{ex'}$ near the target density.

S3 Choosing the contact dielectric constant, with $d = 3$ Å.

As stated in the main article, we explore the idea of local dielectric saturation by implementing the relative dielectric function as a function of r via

$$\varepsilon_r(r) = \begin{cases} \varepsilon_c; & r \leq d \\ \varepsilon_c + (\varepsilon_b - \varepsilon_c)\frac{r-d}{\Delta}; & d < r \leq d + \Delta \\ \varepsilon_b; & r > d + \Delta \end{cases} \tag{15}$$

where $\varepsilon_b = 78.3$ presents the bulk dielectric constant value, $\Delta = 3$ Å is the extent of the linear ramp approximately equalling the diameter of one water molecule as a hydration shell, and ε_c denotes the contact value, selected such that $\varepsilon_c < \varepsilon_b$.

Figure 3 illustrates the onset of phase separation, as ε_c drops below some threshold value (slightly below 22). Figure 3 is actually identical to a corresponding graph the main paper. It is repeated here, in order to make the SI a self-contained document. We note how a decrease of ε_c to values below about 22, produces a long-ranged slope.

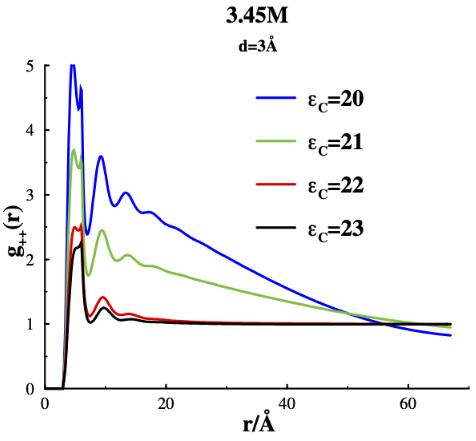

Figure 3: Effect of changing the ε_c on the phase stability of a system with 5000 ion pairs, at 3.45 M. At ε_c below about 22, a steep long-ranged slope is formed.

Such a slope signifies a phase separated system, as illustrated in Figures 4 and 5. They present configuration snapshots from simulations at 3.45 M. VMD was used to produce these illustrations [6, 7]. In Figure 4, we see how a contact value of $\varepsilon_c = 20$ generates pronounced flocculation. In Figure 5, with $\varepsilon_c = 23$, we observe a stable system with significant clustering, but no global phase separation.

S4 Choosing the contact dielectric constant, with $d = 4$ Å.

For completeness, we illustrate that the "optimised value" of ε_c, based on our aim to have an "almost" phase separated system at 3.45 M (having typical experimental saturation values in mind), will depend on the choice of the ion hard-sphere diameter d. Setting $d = 4$Å, we find a similar behaviour as before, as ε_c drops, but the phase transition occurs at a lower value of ε_c. At $\varepsilon_c = 19$ and 20 we obtain a stable single phase but at $\varepsilon_c = 18$ (and below), a long-ranged slope of $g_{++}(r)$ is developed, indicating phase separation. This is shown in Figure 6.

S5 Asymptotic analysis

Within complex analysis of the Fourier transformed Ornstein-Zernike (OZ) equation for a two component mixture, the analytical form of a total correlation function is known [8, 9, 10]

$$rh_{\mu\nu}(r) = \frac{1}{4\pi^2 i} \int_{-\infty}^{\infty} k \exp\left[ikr\right]\hat{h}_{\mu\nu}(k)\, dk \tag{16}$$

where $\mu, \nu \in \{+, -\}$ allow three total correlation functions, $h_{++} \equiv h_{--}$ and h_{+-}. This integral can be analytically solved via complex contour integration, within an infinite semi-circle of the first and second quadrant. The resulting equation

$$rh_{\mu\nu}(r) = \frac{1}{2\pi} \sum_{p\in\mathcal{P}} \exp\left[ik_p{}^{(\mu\nu)}r\right] \mathcal{R}_p{}^{(\mu\nu)} \tag{17}$$

utilises the *Complex Residue theorem*, where $k_p{}^{(\mu\nu)}, p \in \mathcal{P}$ denotes the p-th pole in a set $\mathcal{P}$ of poles (the roots of the OZ equation in k-space), and $\mathcal{R}_p^{(\mu\nu)}$ denotes the complex residue of $k\hat{h}_{\mu\nu}(k)$ at $k_p{}^{(\mu\nu)}$ [8]. It

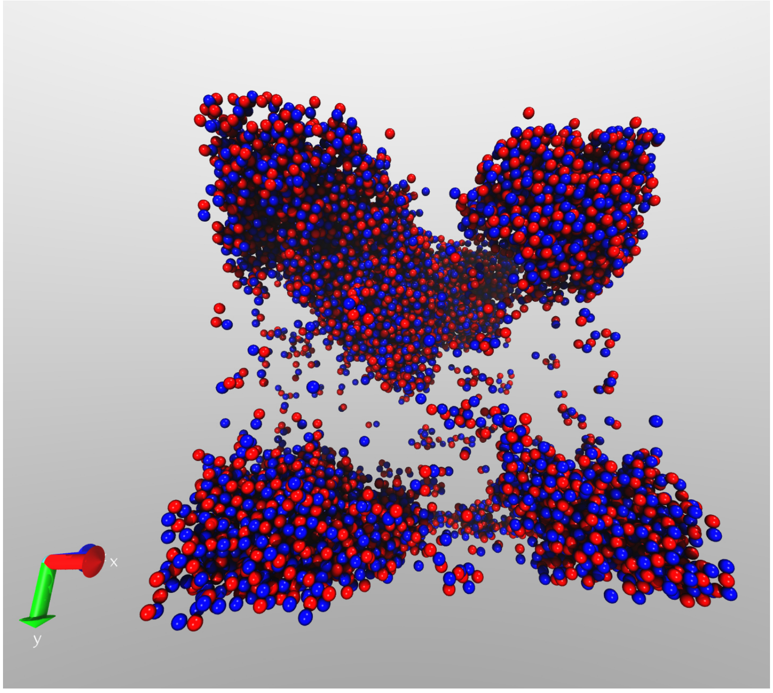

Figure 4: Final configuration snapshot, from a simulation with $\varepsilon_c = 20$, at 3.45 M.

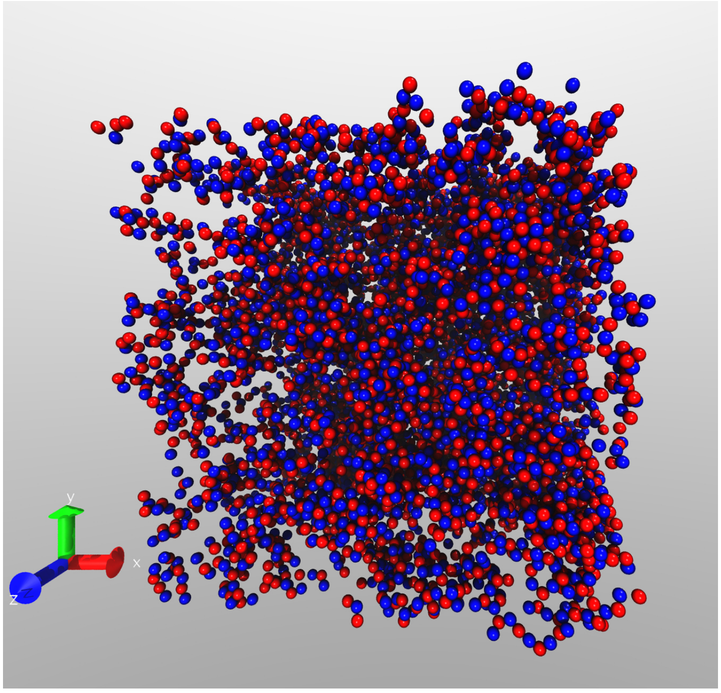

Figure 5: Final configuration snapshot, from a simulation with $\varepsilon_c = 23$, at 3.45 M.

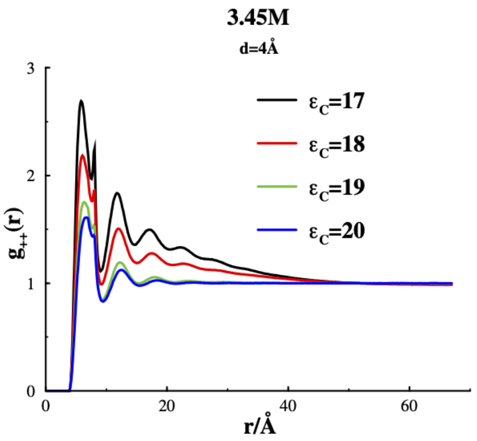

Figure 6: Effect of changing the ε_c on the phase stability of a system with $d = 4$ Å and 5000 ion pairs. At ε_c below about 19, a steep long-ranged slope is formed.

is convenient to define density-density (nn) and charge-charge (zz) total correlation functions via

$$\begin{aligned} h_{nn}(r) &= \frac{1}{4}(h_{++}(r) + h_{--}(r) + 2h_{+-}(r)) \\ h_{zz}(r) &= \frac{1}{4}(h_{++}(r) + h_{--}(r) - 2h_{+-}(r)) \end{aligned} \tag{18}$$

essentially as linear combination of the respective species resolved total correlation functions. Thus by analogy, equation 17 must become a linear combination of the two sets of pole and residue contributions

$$\begin{aligned} rh_{nn}(r) &= \frac{1}{2\pi}\left(\sum_{\mu\in\mathcal{N}} \exp\left[ik_\mu{}^{(nn)}r\right]\mathcal{R}_\mu{}^{(nn)} + \sum_{\nu\in\mathcal{Z}} \exp\left[ik_\nu{}^{(zz)}r\right]\mathcal{R}_\nu{}^{(nn)}\right) \\ rh_{zz}(r) &= \frac{1}{2\pi}\left(\sum_{\mu\in\mathcal{N}} \exp\left[ik_\mu{}^{(nn)}r\right]\mathcal{R}_\mu{}^{(zz)} + \sum_{\nu\in\mathcal{Z}} \exp\left[ik_\nu{}^{(zz)}r\right]\mathcal{R}_\nu{}^{(zz)}\right) \end{aligned} \tag{19}$$

where $\mu \in \mathcal{N}$ indexes the poles of density-density correlations and $\nu \in \mathcal{Z}$ the charge-charge correlations. For the RPM, $h_{++} \equiv h_{--}$ by definition, thus simplifying the equations to [8]

$$\begin{aligned} rh_{nn}(r) &= \frac{1}{2\pi}\sum_{\mu\in\mathcal{N}} \exp\left[ik_\mu{}^{(nn)}r\right]\mathcal{R}_\mu{}^{(nn)} \\ rh_{zz}(r) &= \frac{1}{2\pi}\sum_{\nu\in\mathcal{Z}} \exp\left[ik_\nu{}^{(zz)}r\right]\mathcal{R}_\nu{}^{(zz)} \end{aligned} \tag{20}$$

where each correlation type is determined by the complex poles and residues belonging to the same correlation type. Any given pole of a $(\mu\nu)$ correlation is a complex number with an imaginary component denoted as $\alpha_0^{(\mu\nu)}$ and a real component denoted as $\alpha_1^{(\mu\nu)}$

$$k^{(\mu\nu)} = \alpha_1{}^{(\mu\nu)} + i\alpha_0{}^{(\mu\nu)} \tag{21}$$

Equations 20 reduce to a single dominating pole term with the smallest imaginary component $\alpha_0^{(\mu\nu)}$ in the asymptotic limit, i.e. when $r \to \infty$. For purely complex poles, this asymptotic behaviour is described by an exponential decay of the form

$$rh_{\mu\nu}(r) \sim A^{(\mu\nu)} \exp\left[-\alpha_0^{(\mu\nu)}r\right] \tag{22}$$

and for complex conjugate sets of poles, by a dampened oscillatory decay of the form

$$rh_{\mu\nu}(r) \sim 2\left|A^{(\mu\nu)}\right| \exp\left[-\alpha_0^{(\mu\nu)}r\right] \cos\left(\alpha_1^{(\mu\nu)}r - \theta^{(\mu\nu)}\right) \tag{23}$$

with $A^{(\mu\nu)}$ describing the amplitudes and $\theta^{(\mu\nu)}$ the phase shift originating from the two conjugate real parts of a pair of complex poles. These values can in principle be determined analytically, given analytically obtained total and direct correlation functions (for example by solving the OZ equations with an analytic form for the bridge diagrams) [8]. In practice however, the asymptotic analysis procedure serves as a tool to investigate the asymptotic behaviour of a real (simulated) system.

For a RPM system with competing charge-charge and density-density correlations, two sets of poles exist, $\{k^{(nn)}\}$ and $\{k^{(zz)}\}$. The long-ranged behaviour of a system is dominated by the pole with the smallest imaginary component (α_0), irrespective of the type of correlation [8]. For systems where $\alpha_0^{(nn)} < \alpha_0^{(zz)}$, the total species resolved correlation functions (h_{+-} and h_{++}) behave identically in the long-ranged part, i.e. they approach zero from *above*. If the reverse is true, $\alpha_0^{(nn)} > \alpha_0^{(zz)}$, the total species resolved correlation functions behave as expected for RPM systems, with h_{+-} approaching zero from above and h_{++} from below [8]. On Figure 8, we observe that the $h_{++}(r)$, in grey, transitions from approaching unity from below (for the 0.05 M case) to approaching unity from above, as we increase the concentration. This is indicative of a situation where $\alpha_0^{(nn)}$ becomes the smallest imaginary pole contribution, coinciding with density-density dominated asymptotic behaviour. The sum $h_{++}(r) = h_{nn}(r) + h_{zz}(r)$ directly enables us to analyse the relative contributions from both correlations types, by observing how the grey h_{++} line approaches either the h_{nn} or h_{zz} curves. We observe long-ranged domination of density-density correlations ($h_{++}(r) \sim h_{nn}(r)$), with a short-ranged regime where the dampened oscillatory contribution of h_{zz} produces oscillations superimposed on the simple exponential decay of h_{nn}, Figure 7. At $c > 1$ M we demonstrate h_{nn} asymptotic domination, where the principal decay length is $\alpha_0^{(nn)}$ leading to the functional form

$$rh_{++}(r) = rh_{+-}(r) \sim A^{(nn)} \exp\left[-\alpha_0^{(nn)} r\right] \tag{24}$$

at the asymptotic limit. It is also at this point where charge-charge correlations exhibit a Kirkwood transition from asymptotic exponential decay to asymptotic damped oscillatory decay. Thus, for the RPM model with local dielectric saturation, we observe a long-ranged correlation length which is not an electrostatic or charge-charge correlation length, but rather is a density-density or structural 'cluster' correlation length, described by a simple exponential decay of the form eq. 24.

To contrast the results of the local dielectric constant model, we implemented a renormalised Ornstein-Zernike Hypernetted chain (OZ-HNC) approach [11] to model an RPM system with a constant uniform dielectric constant of 78.3 and 23, Figures 9, 10, 11, and 12. We have previously demonstrated that this approach produces essentially identical results to computer simulations, obtained at a uniform dielectric constant of 78.3, at a much cheaper computational cost [1]. We observe charge-charge domination in all cases, with h_{++} coinciding with h_{zz} for all concentrations when $\varepsilon_r = 78.3$ - see Figure 9. Furthermore, we observe h_{++} approaching zero from below in all cases (Figure 10), which is expected for like-like species resolved total correlation functions for RPM systems. The transition from this behaviour to h_{++} approaching zero from *above*, demonstrated on Figure 8 for c $>$ 50.0 mM for $r > 10$ Å, is a result of density-density correlations, which are orders of magnitude lower for the current model with a uniform bulk dielectric constant, as shown in Figure 9.

For the $\varepsilon_r = 23$ case, the data for 50.0 mM is missing due to convergence issues for dilute systems with strong electrostatic coupling. On Figure 11 we find initial asymptotic domination of charge-charge correlations, but then observe a situation where both charge-charge and density-density correlations contribute in similar parts, (from 0.5 M to 2.0 M). At 3.0 M, the charge-charge exponential dampened oscillatory decay clearly defines the asymptotic behaviour of the system. Thus, we again obtain expected charge-charge domination, even for RPM systems with strong electrostatic coupling (a low value of the uniform dielectric constant).

For completeness, Figures 7, 9, and 11 additionally denote the theoretical Debye screening contribution via $rh(r) \sim \exp\left[-r\lambda_D^{-1}\right]$, dashed line. Comparing with the slopes of $rh_{zz}(r)$ enables us to visually observe anomalous screening behaviour. Similar long ranged slopes describe expected mean-field behaviour, while deviations in the slopes indicate anomalous screening behaviour.

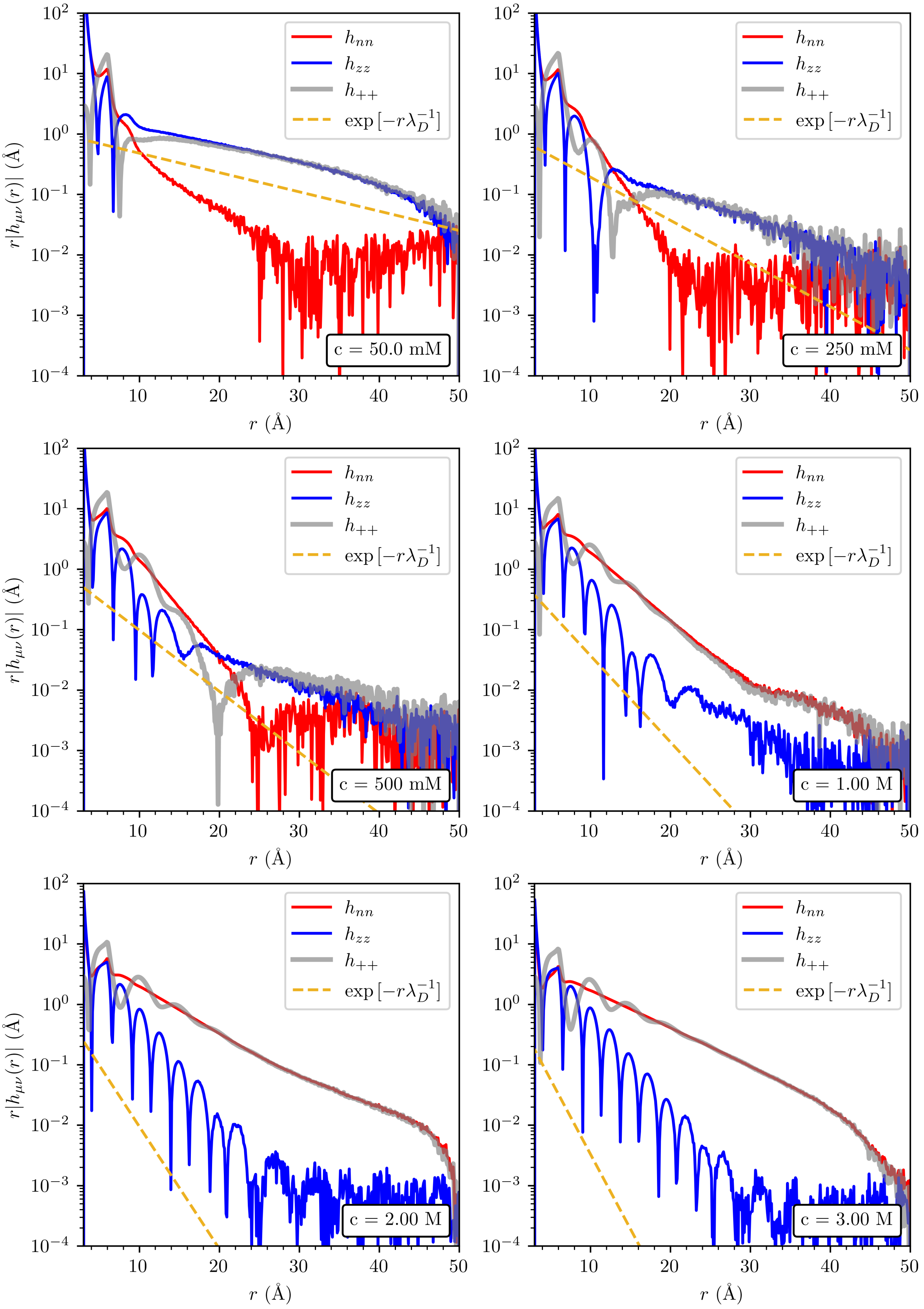

Figure 7: Log scale asymptotic analysis of the total correlation functions for local dielectric saturation. The dashed line denotes the theoretical Debye screening length fit, obtained at a uniform $\varepsilon_r = 78.3$. Note the differences in the slopes of the correlation functions as compared to the Debye fit. Simulation results.

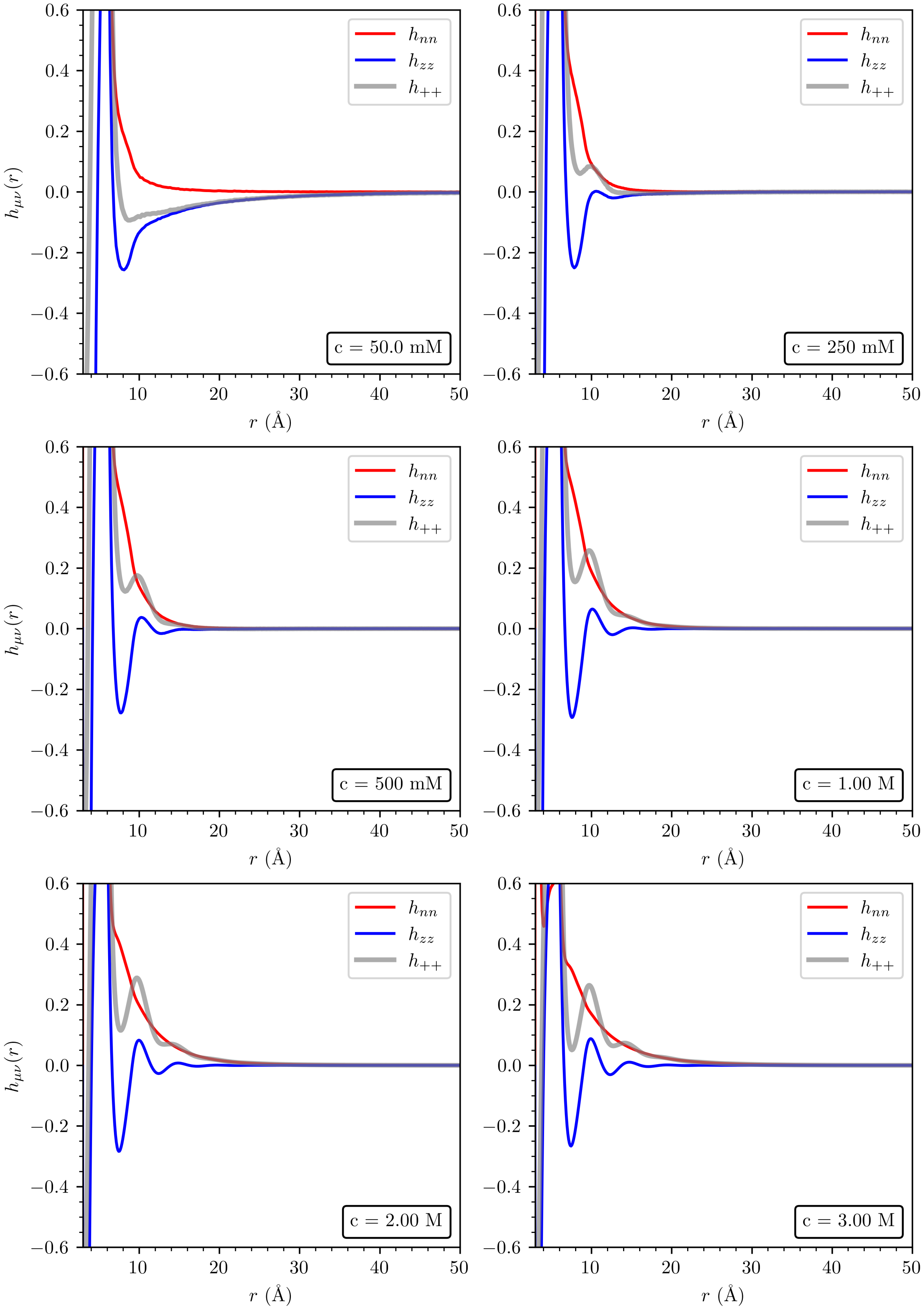

Figure 8: Total correlation functions for local dielectric saturation. Simulation results.

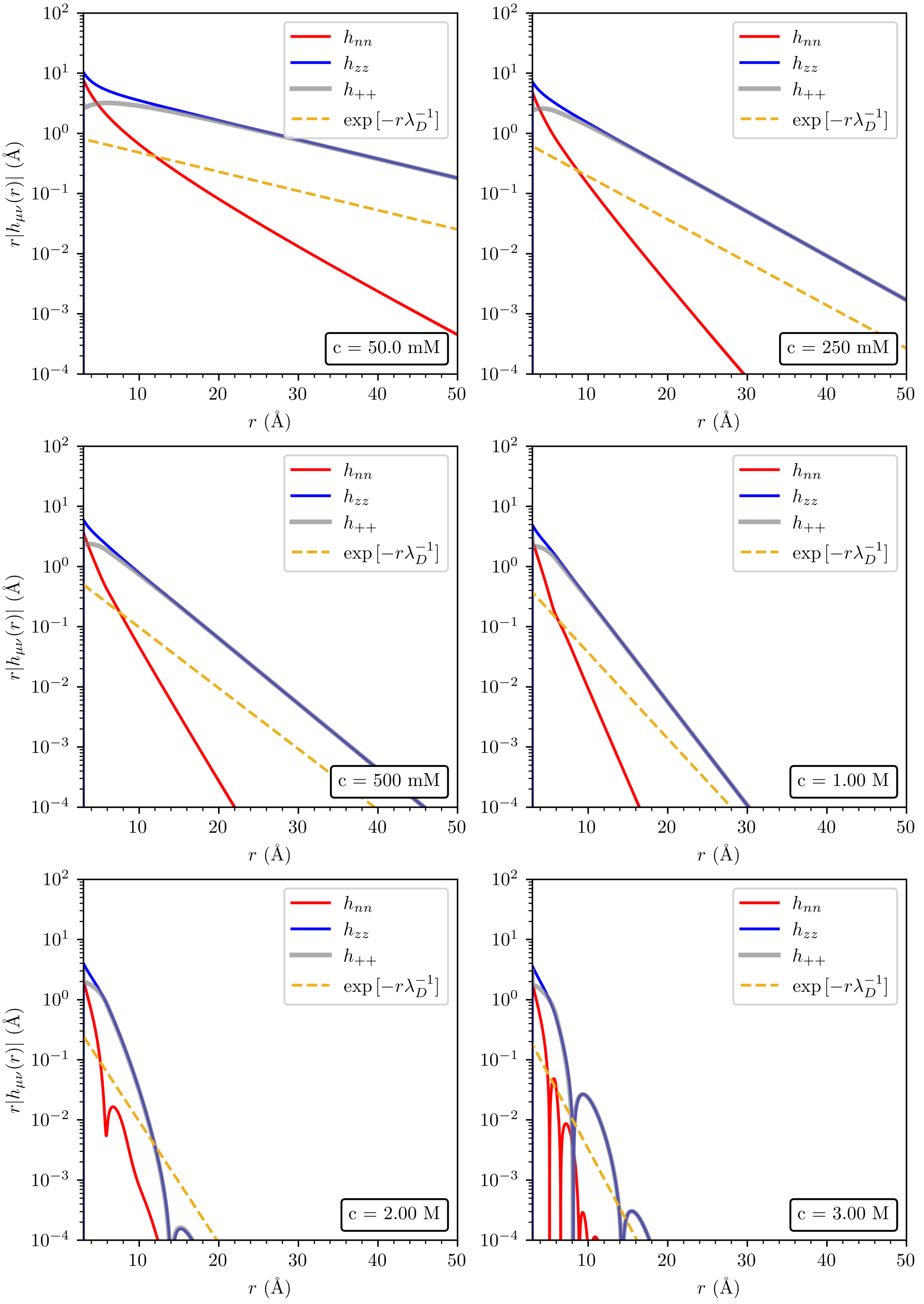

Figure 9: Log scale asymptotic analysis of the total correlation functions for a RPM model with a uniform dielectric constant of $\varepsilon_r = 78.3$. The dashed line denotes the theoretical Debye screening length fit, obtained at a uniform $\varepsilon_r = 78.3$. Note the differences in the slopes of the correlation functions as compared to the Debye fit. OZ-HNC results.

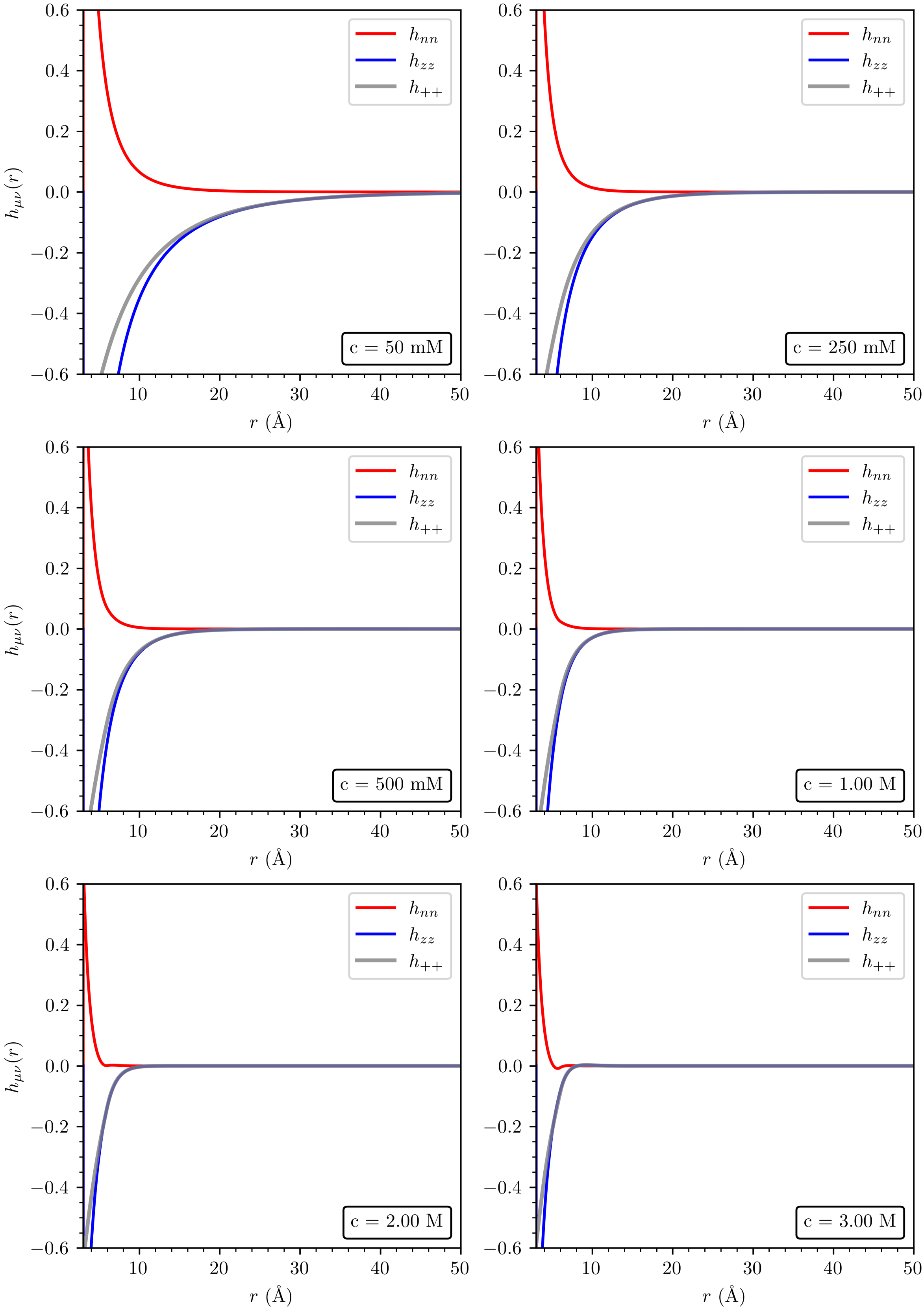

Figure 10: Total correlation functions for a RPM model with a uniform dielectric constant of $\varepsilon_r = 78.3$. OZ-HNC results.

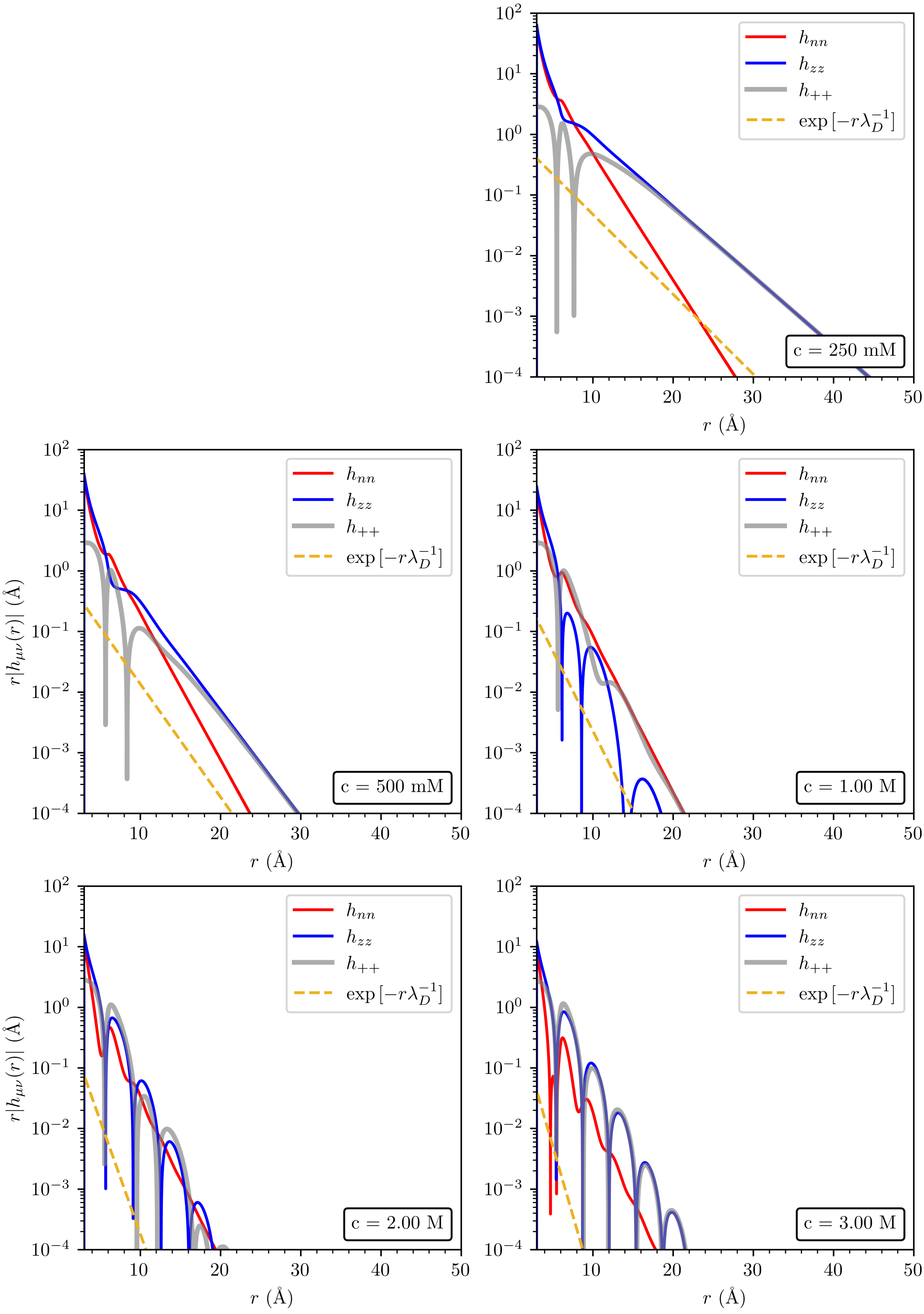

Figure 11: Log scale asymptotic analysis of the total correlation functions for a RPM model with a uniform dielectric constant of $\varepsilon_r = 23.0$. The dashed line denotes the theoretical Debye screening length fit, obtained at a uniform $\varepsilon_r = 23$. Note the differences in the slopes of the correlation functions as compared to the Debye fit. OZ-HNC results. 50.0 mM result not shown due to convergence issues.

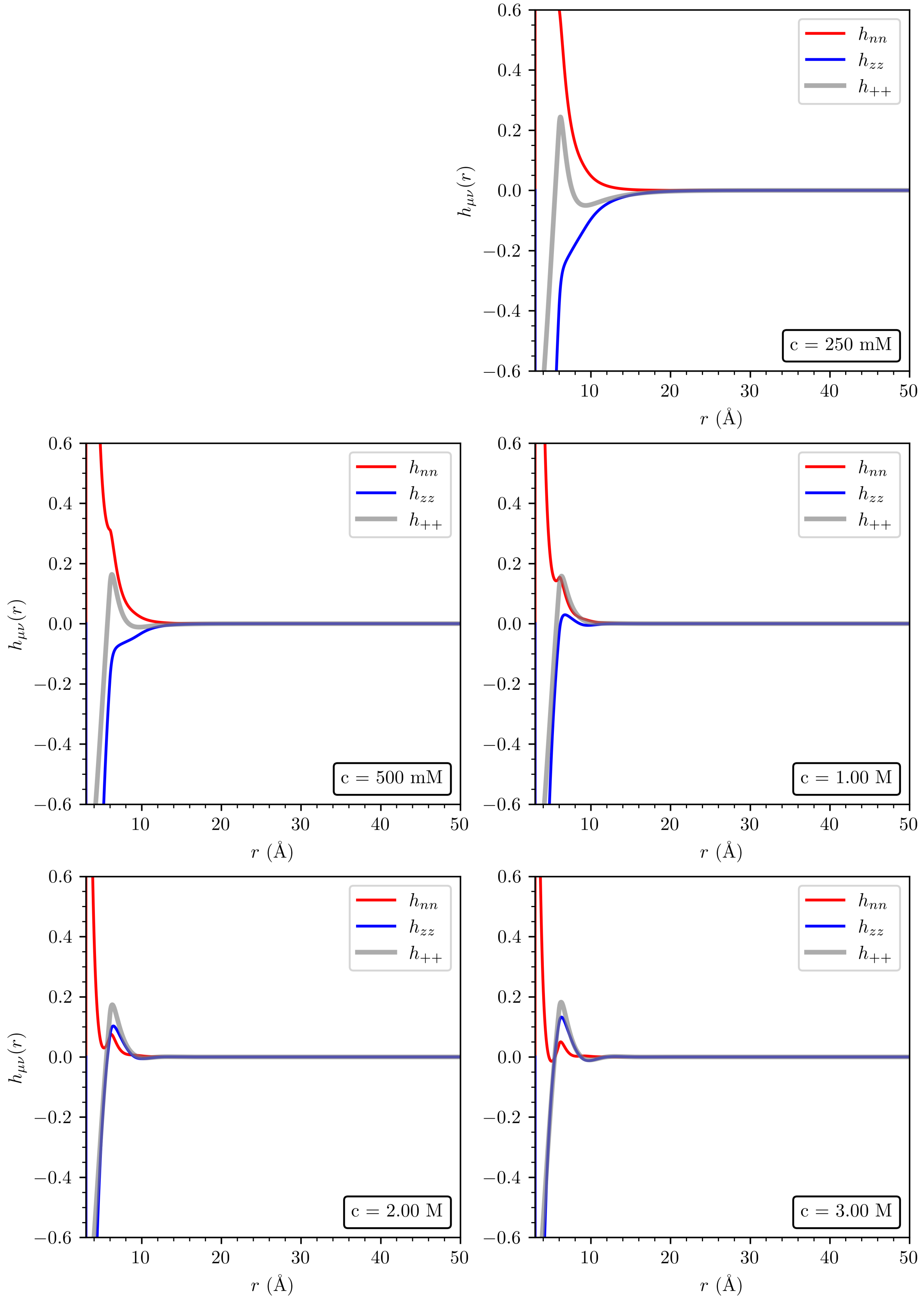

Figure 12: Total correlation functions for a RPM model with a uniform dielectric constant of $\varepsilon_r = 23.0$. OZ-HNC results. 50.0 mM result not shown due to convergence issues.

References

[1] J. Forsman, D. Ribar, C. E. Woodward, An efficient method to establish electrostatic screening lengths of restricted primitive model electrolytes, Physical Chemistry Chemical Physics 26 (2024) 19921–19933. doi:10.1039/d4cp00546e.

[2] B. Jönsson, H. Wennerström, B. Halle, Ion distributions and energetics in lamellar liquid crystals. a comparison between different theoretical approaches, Inorganica Chimica Acta 40 (1980) X39–X40. doi:10.1016/S0020-1693(00)92092-9.

[3] G. M. Torrie, J. P. Valleau, Electrical double layers. i. monte carlo study of a uniformly charged surface, J. Chem. Phys. 73 (1980) 5807–5816. doi:10.1063/1.440065.

[4] K. Shing, K. Gubbins, The chemical potential in dense fluids and fluid mixtures via computer simulation, Molecular Physics 46 (5) (1982) 1109–1128. doi:10.1080/00268978200101841.

[5] P. Sloth, T. S. Sörensen, Monte carlo calculations of chemical potentials in ionic fluids by application of widom's formula: Correction for finite-system effects, Chemical Physics Letters 173 (1) (1990) 51–56. doi:10.1016/0009-2614(90)85301-R.

[6] W. Humphrey, A. Dalke, K. Schulten, VMD – Visual Molecular Dynamics, Journal of Molecular Graphics 14 (1996) 33–38.

[7] J. Stone, *An Efficient Library for Parallel Ray Tracing and Animation*, Master's thesis, Computer Science Department, University of Missouri-Rolla (April 1998).

[8] R. Leote de Carvalho, R. Evans, The decay of correlations in ionic fluids, Molecular Physics 83 (4) (1994) 619–654. doi:10.1080/00268979400101491.

[9] M. E. Fisher, B. Wiodm, Decay of correlations in linear systems, The Journal of Chemical Physics 50 (9) (1969) 3756–3772. doi:10.1063/1.1671624.

[10] P. Attard, Asymptotic analysis of primitive model electrolytes and the electrical double layer, Phys. Rev. E 48 (1993) 3604–3621. doi:10.1103/PhysRevE.48.3604.

[11] T. Ichiye, A. D. J. Haymet, Accurate integral equation theory for the central force model of liquid water and ionic solutions, The Journal of Chemical Physics 89 (7) (1988) 4315–4324. doi:10.1063/1.454815.
